# An Updated Risk Assessment as Part of the QbD-Based Liposome Design and Development

**DOI:** 10.3390/pharmaceutics13071071

**Published:** 2021-07-13

**Authors:** Zsófia Németh, Edina Pallagi, Dorina Gabriella Dobó, Gábor Kozma, Zoltán Kónya, Ildikó Csóka

**Affiliations:** 1 Faculty of Pharmacy, Institute of Pharmaceutical Technology and Regulatory Affairs, University of Szeged, 6. Eötvös u, H-6720 Szeged, Hungary; nemeth.zsofia@szte.hu (Z.N.); pallagi.edina@szte.hu (E.P.); dobo.dorina.gabriella@szte.hu (D.G.D.); 2 Department of Applied and Environmental Chemistry, Faculty of Science and Informatics, Institute of Chemistry, University of Szeged, 1, Rerrich Béla tér, H-6720 Szeged, Hungary; kozmag@chem.u-szeged.hu (G.K.); konya@chem.u-szeged.hu (Z.K.)

**Keywords:** Quality by Design, initial risk assessment, updated risk assessment, critical factors, “intermediate” liposome formulation, thin-film hydration method, liposome characterisation

## Abstract

Liposomal formulation development is a challenging process. Certain factors have a critical influence on the characteristics of the liposomes, and even the relevant properties can vary based on the predefined interests of the research. In this paper, a Quality by Design-guided and Risk Assessment (RA)-based study was performed to determine the Critical Material Attributes and the Critical Process Parameters of an “intermediate” active pharmaceutical ingredient-free liposome formulation prepared via the thin-film hydration method, collect the Critical Quality Attributes of the future carrier system and show the process of narrowing a general initial RA for a specific case. The theoretical liposome design was proved through experimental models. The investigated critical factors covered the working temperature, the ratio between the wall-forming agents (phosphatidylcholine and cholesterol), the PEGylated phospholipid content (DPPE-PEG_2000_), the type of the hydration media (saline or phosphate-buffered saline solutions) and the cryoprotectants (glucose, sorbitol or trehalose). The characterisation results (size, surface charge, thermodynamic behaviours, formed structure and bonds) of the prepared liposomes supported the outcomes of the updated RA. The findings can be used as a basis for a particular study with specified circumstances.

## 1. Introduction

According to the European Medicine Agency (EMA), “liposomes are classically described as artificially prepared vesicles composed of one or more concentric lipid bilayers enclosing one or more aqueous compartments” [1]. These vesicles can be described as “microscopic phospholipid bubbles with a bilayered membrane structure” and an aqueous media in the centre [2]. In this way, liposomes provide a suitable delivery system for both the hydrophobic drugs (in the membrane) and the hydrophilic compounds (in the central part).

A. D. Bangham developed the first liposomes in the early 1960s [3]. Since that time, liposomes have been proved to be successful nanocarriers for a targeted gene and drug delivery; however, as the amount of information and improvements on liposomes increases, the scale of the challenges in the field raises as well. To get a high-quality product, knowledge of medical, pharmaceutical, chemical, biological and physical sciences should be used and mixed with technological experiences [4]. All this information needs to be considered, organised and evaluated to achieve a successful liposome-based formulation development [5].

The Quality by Design (QbD) concept is a knowledge-, and risk assessment-based quality management approach, used mainly in the pharmaceutical industrial production process [6,7]; however, it also can be extended and applied in the early pharmaceutical research and development (R&D) phase [5,8,9]. Nowadays, including the QbD elements during the submissions of the marketing authorisation documents is a regulatory requirement. The QbD is a holistic and systemic way of improvements, where the primary focus is on the profound preliminary target product design. Thus, the theoretical design phase is extended based on prior knowledge (from literature and previous research) and risk estimation. This accurate design, especially the implementation of the Risk Assessment(s) (RA), helps correctly set up the practical experiments.

The whole method and the elements of the QbD are described in the guidelines of the International Council for Harmonisation of Technical Requirements for Pharmaceuticals for Human Use (ICH) [10,11,12].

A QbD method-guided development has several steps that are specified in the guidelines mentioned above. The first step is the definition of the Quality Target Product Profile (QTPP), which contains the essential parameters of the formulation from the patient’s point of view and the requirements from the clinical field. The QTPP is a prospective summary of the quality characteristics of the product that ideally will be achieved. It is related to the quality, safety and efficacy of the product, considering, for example, the route of administration, the dosage form, the bioavailability, the strength and the stability. [10]. The QTPP selection is followed by the design of the product and the manufacturing process according to the predefined quality profile, which means selecting those parameters that have a critical influence on the QTPP. These are the Critical Quality Attributes (CQAs), which are related to the safety and efficacy of the product. The CQAs are those physical, chemical, biological or microbiological properties or characteristics that should be within an appropriate limit range or distribution to ensure the targeted product quality [10]. The potential CQAs of the drug product are derived from the QTPPs, and prior knowledge guides the product and process development [10]. Other crucial factors are the Critical Material Attributes (CMAs) related to the materials and the Critical Process Parameters (CPPs) associated with the selected production method. The CPPs are those factors that should be monitored or controlled to ensure the process maintains the aimed quality [10]. The key element of a QbD-guided development is the RA (initial, recurrent/updated or finalised) [6]. This process results in the CQAs/CPPs ranked by their critical effect on the targeted product quality. Then, the Design of Experiments (DoE) [10] can be set up based on the results of the RA, which means that the practical experiments are planned and carried out according to the most relevant influencing factors (CMAs and CPPs). In the next step, the determination of the Design Space (DS) [13] of the product can be performed. The DS has remarkable regulatory benefits because the alterations in the production parameters in the DS do not require modifications during the submission. The following steps of the QbD method are the application of the Control Strategy and the planning of the Continuous Improvement, which have relevance from the perspective of the pharmaceutical industry. The QbD-, and RA-based development and screening have several advantages; thus, the experiments could be more effective in practice, and it can be especially useful in the early pharmaceutical developments of complex or sensitive drugs or systems with special considerations [14,15,16,17,18,19,20,21,22].

The requirements for the liposomal formulations vary depending on the chosen medical need and the selected route of administration. The proper liposome formulation design is assigned to the therapeutic needs. Furthermore, identifying and collecting those factors that impact the final product is an essential step. The factors that critically influence the quality and characteristics of the liposomes require the most significant attention during the development process. The critical parameters affecting the liposomes were collected and evaluated both in general and with particular attention to the process parameters of the thin-film hydration method in a previous work from our research group (initial RA) [5] to extend the QbD method to the early development phase of the liposome-related pharmaceutical researches. A general overview of the QbD approach for liposomes without a defined production process completed with characterisation methodologies is available, owing to the work made by Porfire et al. [23]. Xu et al. performed a risk analysis study on liposomes gained from the thin-film hydration technique and loaded with superoxide dismutase via a freeze-thaw cycling technique [24]. Their evaluation involved the analytics and the instrumentation reliability as well. The findings of the previously mentioned studies were built into our former theoretical article [5]. Ahmed et al. combined the QbD tools with process analytical technology to support the development of transdermal glimepiride liposomal films [25]. The results of the risk analysis were studied according to the design by Plackett–Burman. Factorial design-based RA results were utilised to evaluate the formulation variables of an early development phase, nose-to-brain, lipophilic API-containing liposome preparation by Pallagi et al. [26]. Chitosan-coated ghrelin-containing liposomes were developed for intranasal delivery following the RA steps by de Barros et al. [27] to determine the optimal active pharmaceutical ingredient (API) and chitosan concentrations applying the thin-film hydration method. Pandey et al. applied the QbD method in the development of chitosan-coated, hydrophilic API-enclosing liposomes prepared via a modified ethanol injection method [28]. Merlo-Mas et al. presented the use of the QbD tools and risk analysis in the case of the α-galactosidase-loaded nanoliposomes preparation through the DELOS-susp (depressurisation of an expanded liquid organic solution into aqueous solution) method, a compressed fluid-based technique that results in reproducible and scalable nanovesicular systems [29]. As the presented list about the diversified applications of the QbD approach shows, this quality management method is useable when complex fields need to be combined to meet the development, therapeutic, authorisation and patient-centred requirements.

This present research aimed to investigate the critical parameters highlighted in the initial RA [5] from new perspectives, carry out a comparative characterisation study and determine the general effects of the selected CMAs and CPPs on the properties of the liposomes. Accordingly, an evaluation to perform an updated RA was targeted for “intermediate” API-free liposomal formulations to get a practical decision-making system that enables the change of the liposome properties according to possible predefined goals (i.e., requirements of the API, the dosage form and the administration route) in the future.

## 2. Materials and Methods

### 2.1. Materials

Two different compositions were used to form liposomes, with modifications in the phospholipid and cholesterol ratios according to the goal of the investigations. One of the compositions was a phosphatidylcholine- and cholesterol-based simple formulation (hereinafter: PC-CH, Table 1 and Table 3), while the other one contained PEGylated phosphatidylethanolamine as well (hereinafter: PC-CH-PEG, Table 2 and Table 3).

The following materials were used as liposomal wall-forming excipients (in an alcoholic solution): cholesterol (CH) (Molar Chemicals Kft., Budapest, Hungary), L-α-phosphatidylcholine (PC) (Sigma-Aldrich Chemie GmbH, Munich, Germany) and 1,2-dipalmitoyl-sn-glycero-3-phosphoethanolamine-N-[methoxy(polyethylene glycol)-2000] (ammonium salt) (DPPE-PEG_2000_) (Avanti^®^ Polar Lipids Inc., Alabaster, Alabama, AL, USA), solved in ethanol 96% (Molar Chemicals Kft., Budapest, Hungary). The excipients were used in different ratios (Table 1 and Table 2).

Phosphate-buffered saline pH 7.4 (PBS pH 7.4) and pH 5.6 (PBS pH 5.6) and sodium chloride physiological solution (saline solution) pH 5.5 [30] were used as hydration media. The composition of these solutions are the followings: PBS pH 7.4: 8.0 g/L NaCl, 0.20 g/L KCl, 1.44 g/L Na_2_HPO_4_ × 2 H_2_O, 0.12 g/L KH_2_PO_4_; PBS pH 5.6: 0.65 g/L K_2_HPO_4_, 8.57 g/L KH_2_PO_4_; saline solution: 9.0 g/L NaCl dissolved in distilled water. The materials used to make these hydration media are the following: sodium chloride (NaCl) (Molar Chemicals Kft., Budapest, Hungary), potassium chloride (KCl) (Molar Chemicals Kft., Budapest, Hungary), disodium hydrogen phosphate dihydrate (Na_2_HPO_4_ × 2 H_2_O) (Spektrum-3D Kft., Debrecen, Hungary), dipotassium phosphate (K_2_HPO_4_) (Spektrum-3D Kft., Debrecen, Hungary) and potassium dihydrogen phosphate (KH_2_PO_4_) (Molar Chemicals Kft., Budapest, Hungary). Three carbohydrates were used as cryoprotectants for lyophilisation, such as d-glucose (Hungaropharma Zrt., Budapest, Hungary), d-sorbitol (Hungaropharma Zrt., Budapest, Hungary) and d-trehalose (Sigma-Aldrich Chemie GmbH, Munich, Germany). The amount of the chosen cryoprotectant was 5% of the phospholipid mass in every case, solved in the hydration media [31]. None of the formulations contained active pharmaceutical ingredients (API).

### 2.2. Methods

#### 2.2.1. Elements of the QbD Design

##### Development of the Knowledge Space and Determination of the QTPP

Determining the QTPP of the aimed formula is the essential first step in the QbD-guided development process. For this purpose, a primary knowledge space development [15] must be carried out, which means collecting and systematising all the relevant information regarding the aimed product and the production. Besides the definition of the QTPP, this step can help identify the potential critical factors of the formulation development. In this case, an “intermediate” API-free liposomal product was targeted as the QTPP, with the following requirements: spherical, large unilamellar vesicles (LUVs) in stable, monodisperse systems. Homogeneity was the requirement for the liposome formulations in an aqueous solution form and a dry solid phase for the lyophilised powders. 

##### Determination of the CQAs, the CMAs and the CPPs

The nomination of a factor as a CQA, CMA or CPP always depends on the predefined goals, the expected quality of the product, the therapeutic needs and the selected production process. The stability, the zeta potential, the size of the vesicles, the number of lamellas, the polydispersity index, the surface modifications (in this study: PEGylation) and the phase transition temperature were identified as the CQAs, and the following factors were enumerated in the CMAs/CPPs group: the quality of the phospholipids and the cholesterol derivatives, the ratio between the phospholipids and the cholesterol, the surface modifiers, the phase transition temperature of the lipids, the quality of the solvent, the hydration media and the cryoprotectants, the working temperature and the process settings

##### Risk Assessment

After identifying the risks, the LeanQbD^®^ software (QbD Works LLC, Fremont, CA, USA) was used for the RA process. The first element of this procedure was the interdependence rating between the QTPPs and the CQAs and the CQAs and CMAs/CPPs. A three-level (1-3-9) scale was used to describe the relationship between the parameters as “high” (H), “medium” (M) or “low” (L) and the results presented in Risk Estimation Matrices. Then, a risk occurrence rating (or probability rating step) was made for the CMAs/CPPs, using the same three-grade scale (H/M/L) for the analysis. The scoring was done for each parameter pair individually. After the scoring, the combination of the information provided a risk evaluation transforming the established risk levels into numerical scorings [32]. As the output of the RA evaluation, Pareto diagrams [33] were generated by the software presenting the numeric data and the ranking of the CQAs and CMAs/CPPs according to their potential impact on the aimed final product (QTPP). Due to that generated origin of the severity scores (which is influenced by the level number of the scale used for the analysis), the relative position of the factors should be considered instead of their value. The Pareto charts show the differences between the effects of the CMAs and the CPPs and help select the experimental design factors. 

##### Design of the Experiments

Based on the results of the RA, the DoE was built up. Five variables were identified as CMAs/CPPs: the working temperature, the phosphatidylcholine:cholesterol mass ratio, the PEGylated phospholipid content, the quality of the hydration media and the quality of the cryoprotectants. Each factor was investigated at different levels (Table 4). The effect of the working temperature and the phosphatidylcholine:cholesterol mass ratio on the PC-CH composition was investigated. Using the information obtained from these early studies, the effect of the PEGylated phospholipid content, the quality of the hydration media and the type of the cryoprotectant were investigated under improved conditions (pre-set temperature (60 °C) and phospholipid:cholesterol mass ratio (60:40)) on the PC-CH-PEG formulations. Three parallel samples were made and checked for each measurement.

#### 2.2.2. Preparation of Liposomes and Process of Lyophilisation

The preparation of the liposome samples was based on the thin-film hydration method [34]. This method ensures a stable and straightforward way for liposome preparation [35] and, according to previous experiences [26], can be easily adapted for liposome studies. The alcoholic solutions of the wall-forming agents were used in the optimised concentrations regarding the chosen formulations (PC-CH or PC-CH-PEG). The ethanol was evaporated in a water bath under decreasing pressure with the Rotavapor^®^ R-210/215 (BÜCHI Labortechnik AG, Flawil, Switzerland) rotary evaporator. The rotation speed was 25 rpm. Firstly, the temperature of the water bath was investigated, and the preparations were done at 50 °C, 60 °C and 70 °C; then, 60 °C was chosen and used for the later formulations. The decrease in air pressure was gradual. The pressure was decreased with steps of 100 mbar and kept at the lowest value (100 mbar) while an entire film has formed. The dried lipid film was hydrated with selected hydration media. The formation of the vesicles was supported by ultrasonication (Elmasonic S 30 H ultrasonic bath, Elma Schmidbauer GmbH, Singen, Germany). The sonication was performed at the investigated temperature for 30 min. The shaping of the liposomes happened in two steps via vacuum membrane filtration using a 0.45 µm (nylon membrane disk filter 47 mm, Labsystem Kft., Budapest, Hungary), then a 0.22 µm membrane-filter (Ultipor^®^ N66 nylon 6.6 membrane disk filter 47 mm, Pall Corporation, New York, NY, USA). The vacuum was created by a vacuum pump (Rocker 400 oil-free vacuum pump, Rocker Scientific Co., Ltd. New Taipei City, Taiwan). The prepared liposome samples were immediately investigated for vesicle size, polydispersity and zeta potential; some samples were stored in liquid state and retested, but all were lyophilised for further investigations. The lyophilisation was done via SanVac CoolSafe freeze dryer (LaboGeneTM, Lillerød, Denmark) at normal atmospheric pressure, gradually decreasing the temperature from +25 °C to −40 °C. The vacuum was created when the temperature of the samples reached the chosen value, reducing the pressure to 0.01 atmosphere where the samples were stored for 8–10 h. After this period, the temperature of the tray was increased manually step by step from −40 °C to +25 °C until the pressure reached the normal atmospheric value. The lyophilised samples were stored in closed vials at 2–8 °C.

#### 2.2.3. Characterisation of the Liposomes

##### Vesicle Size and Zeta Potential Analysis

Dynamic light scattering (DLS) technique was used to determine the vesicle size (expressed in Z-average) and the polydispersity index (PdI), referring to the heterogeneity or uniformity of the particles in the investigated samples. For the lipid-based nanocarrier systems, PdI values less or equal to 0.3 are considered the indicator of a monodisperse distribution [28]. The studied samples were accepted as a suitable formulation around or below this value. 1 mL was investigated from each sample in folded capillary zeta cells (Malvern Panalytical Ltd., Malvern, Worcestershire, UK). Zeta potential is the potential difference between the investigation media and the stationary fluid layer adsorbed to the surface of the particles, and among others, describes the stability of a formulation. Low zeta potential values indicate the aggregation of the dispersed particles, while higher potentials refer to a more stable formulation [36]. Vesicles with a charge less or equal to 10 mV are considered negatively, more or equal to 10 mV as positively charged, while between these two values as neutral liposomes [37]. These values were measured via the Malvern Zetasizer Nano ZS system (Malvern Panalytical Ltd., Malvern, Worcestershire, UK), equipped with a 633 nm wavelength laser.

##### Differential Scanning Calorimetry (DSC) and Thermogravimetric Analysis (TGA) Investigations

The thermodynamic state of the liposomes was studied in the temperature range of 25–300 °C via differential scanning calorimetry (DSC) technique (TA Instruments DSC Q20, TA Instruments, New Castle, Delaware, DE, USA). The number of the possible phase transitions (T_m_) and the gel to liquid-crystalline phase transition temperature (T_c)_ were determined using a 10 °C/min heating rate. Freeze-dried samples of 6–10 mg were studied and placed into hermetically sealed aluminium sample pans in dry nitrogen gas. During the thermogravimetric (TGA) measurements, the lyophilised samples are heated to a defined temperature and investigated for mass changes. The Setaram Labsys TG-DTG-DTA analyser (SETARAM Instrumentation, Caluire, France) was used for the investigations. The studies were done in a nitrogen atmosphere in the temperature range of 25–300 °C with a 10 °C/min heating rate from 8–10 mg freeze-dried samples.

##### Fourier-Transform Infrared (FT-IR) Spectroscopy Measurements

The interactions between the compounds of the liposome products were measured via an Avatar 330 FT-IR Thermo Nicolet spectrometer (Thermo Electron Corporation, Waltham, MA, USA) equipped with an infrared light source and optics. The measurements were made from freeze-dried powder samples in 4000–400 cm^−1^ wavelength range with 4 cm^−1^ spectral resolution in absorbance mode. For sample preparation, the lyophilised powders were mixed with potassium bromide (KBr), pulverised and pressed to form pellets. KBr pellets were used as references.

##### Atomic Force Microscopy (AFM) Measurements

In this investigation, one drop of the formulation was applied on a freshly cleaved mica surface (Muscovite mica, V-1 quality, Electron Microscopy Sciences, Washington, DC, USA) to obtain AFM images under normal ambient conditions using the tapping mode of an NT-MDT SolverPro Scanning Probe Microscope (NT-MDT, Spectrum Instruments, Moscow, Russia). AFM tips type PPP-NCHAuD-10 (thickness: 4.0 μm, length: 125 μm, width: 30 μm) (NanoWorld AG, Neuchâtel, Switzerland) was applied with 2 nm nominal radius of curvature and 15 μm length. The non-contact silicon cantilevers had a typical force constant of 42 N/m and a resonance frequency of 330 kHz. 

##### Residual Ethanol Measurements via Gas Chromatography-Mass Spectrometry (GC-MS)

The determination of the residual ethanol content of the samples was carried out using a Shimadzu GCMS-QP2010 SE gas chromatograph-mass spectrometer (Shimadzu Corporation, Kyoto, Japan). A total of 50 mg from the freeze-dried samples were sonicated in 1 mL toluene, settled, decanted and filtered through a 0.22 μm polytetrafluoroethylene syringe filter (Thermo Fisher Scientific Inc., Waltham, MA, USA). A sample of 1 μL was investigated. The oven program was as follows: initial temperature 80 °C for 2 min, increased at 20 °C/min to 180 °C, held at 180 °C for 2 min. The mass spectrometer measured from 0.5 min to 1.6 min, and from 25 m/z to 46 m/z with continuous scan.

#### 2.2.4. Statistical Analysis

Data analysis, statistics and graphs were performed from the experimental data via Microsoft^®^ Excel^®^ (Microsoft Office Professional Plus 2013, Microsoft Excel 15.0.5023.100, Microsoft Corporation, Redmond, WA, USA), OriginPro^®^ 8.6 software (OriginLab^®^ Corporation, Northampton, MA, USA) and JMP^®^ 13 Software (SAS Institute, Cary, NC, USA). The significance of the difference between a pair of investigated formulation groups was calculated via a one-way analysis of variance (ANOVA) with post-hoc Tukey test in Minitab^®^ 17.1.0 software (Minitab, LLC, State College, Pennsylvania, PA, USA) with *p* < 0.05 as a minimal level of significance. Results were expressed as the mean value ± standard error. Three independently prepared parallel samples were made and studied in the investigations.

## 3. Results

### 3.1. Development of the Knowledge Space, the Definition of the QTPP and the Identification of the CQAs, CMAs and CPPs

Based on the quality-concerned requirements of the liposomal formulations [5], the relevant properties were narrowed down, and a stable, LUV-containing, monodisperse and homogeneous, API-free formulation was determined as the QTPP of the “intermediate” liposomal products that can provide a base for later carrier systems (Table 5). The elements and factors that can be identified as the CQAs of the aimed liposome preparation are shown in detail in Table 6. These specified CQAs include the size of the vesicles, the number of the lamellas, the polydispersity index, the zeta potential, the stability, the surface modifications (PEGylation) and the phase transition temperature of the liposomes. The CMAs and the CPPs are the quality of the phospholipids and the cholesterol derivatives, the ratio between the wall forming agents, the surface modifiers, the phase transition temperature of the lipids, the quality of the solvent, the hydration media, the cryoprotectants and further additives, the working temperature, the sterility requirements and the settings of the thin-film hydration method (dissolution, vacuum evaporation, sonication, filtration, lyophilisation and storage).

### 3.2. Risk Assessment

**Figure 1:**
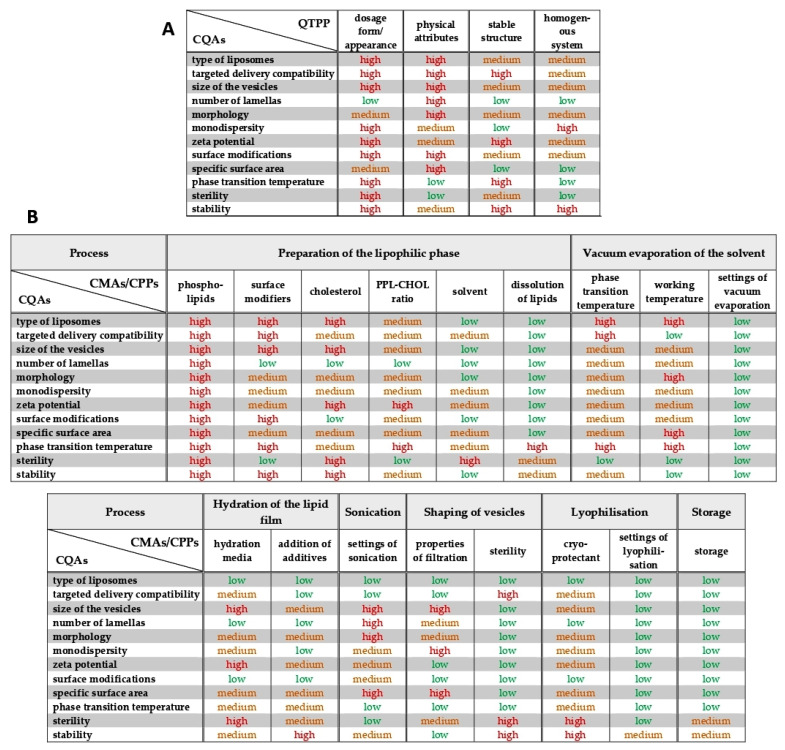
Results of the interdependence investigations between the elements of the Quality Target Product Profile (QTPP) and the Critical Quality Attributes (CQAs) (**A**), and between the CQAs and the Critical Material Attributes (CMAs) and the Critical Process Parameters (CPPs) (**B**).

**Figure 2:**
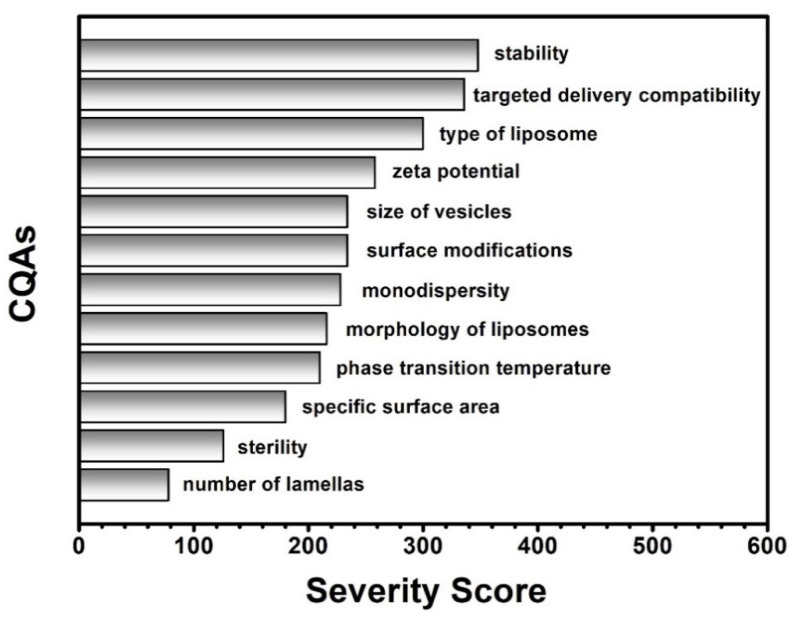
List of the Critical Quality Attributes (CQAs) of the liposomes ranked by their calculated severity scores.

**Figure 3:**
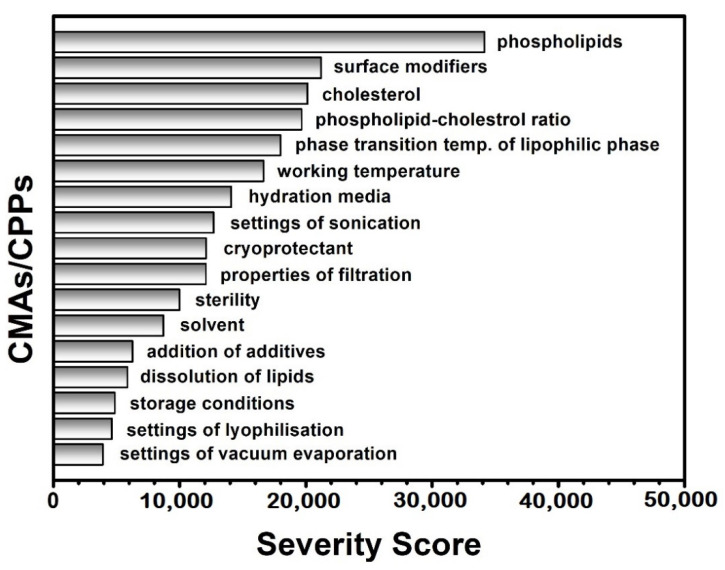
List of the Critical Material Attributes (CMAs) of the liposome components and the Critical Process Parameters (CPPs) of the thin-film hydration preparation method ranked by their calculated severity scores.

After the profound and careful knowledge space development and the determination of the QTPP and the CQAs of the potential liposome carriers presented above, the classification of the CMAs and CPPs by their criticality was performed during the risk analysis. A research group-level brainstorming utilising the prior experiences and the literature knowledge supported the three-grade scaled interdependence rating between the items of the QTPP elements and the CQAs and between the CQAs and CMAs/CPPs, helping to determine the severity of the risks what the factors mean to each other (Figure 1). The sonication has a strong impact on the lamellarity and the size of the liposomes (interdependence evaluated as “high”), while it does not influence the phase transition temperature of the lipid formulation (effect estimated as “low”) (see in Figure 1). Based on the Risk Estimation Matrices and the results of the occurrence rating of the factors, the software transformed the given data into numerical information and calculated the overall severity of the risks. The generated Pareto charts show the ranking of the critical factors, presented in Figure 2 and Figure 3. 

According to the RA process, the CMAs/CPPs are the followings organised in descending order based on their criticality: quality of phospholipids, quality and quantity of surface modifiers, ratio between the phospholipids and the cholesterol, cholesterol content, phase transition temperature, working temperature, quality of the hydration media, settings of sonication, quality and quantity of cryoprotectants, properties of filtration, sterility, quality of solvent, addition of additives, dissolution of lipids, storage conditions, settings of lyophilisation and vacuum evaporation. The settings of the thin-film hydration process were kept in formerly set stable values, except the working temperature, which was chosen for further investigation based on its high severity score. The phospholipid:cholesterol ratio, the effect of a PEGylated phospholipid as the equivalent of the surface modifications in this study and the quality of the hydration media and the cryoprotectant were investigated from the relevant CMAs. The quality of the solvent was set as ethanol 96% in all the experiments. The factors of the CMAs/CPPs were studied according to the DoE (Table 4).

### 3.3. Characterisation Results of the Liposomal Products

The following data show how the changes in some of the CPPs (working temperature) and CMAs (phospholipid:cholesterol mass ratio, addition of PEGylated phospholipids, quality of the hydration media and quality of the cryoprotectants) affect the characteristics of the API-free liposomal products. Different derivatives of the two basic formulations (PC-CH and PC-CH-PEG) were used for the investigations and characterised.

#### 3.3.1. Effects of Using Different Temperature Values

**Figure 4:**
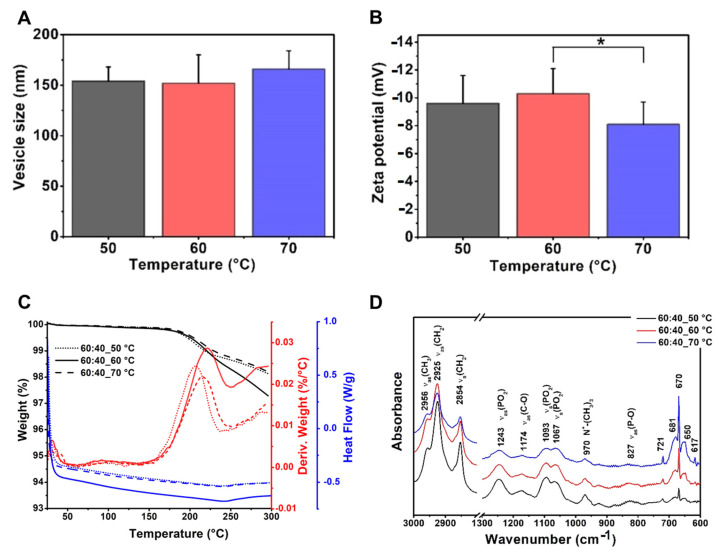
Characteristic features of the liposome samples prepared at different temperature values (50, 60 and 70 °C) from PC-CH 60:40 mass ratio composition, and hydrated with saline solution, presenting the results of the investigations: vesicle size and zeta potential analysis (**A**,**B**), differential scanning calorimetry and thermogravimetric analysis (**C**) and Fourier-transform infrared spectroscopy (**D**). *: *p* < 0.05.

The effect of the working temperature is a little-investigated factor regarding the size of the liposomes. In this research, phosphatidylcholine–cholesterol (mass ratio: 60:40) vesicles were prepared at 50, 60 and 70 °C. The mean vesicle size values were measured as 154–166 nm with no significant difference (Table 7, Figure 4). The polydispersity indexes were under the acceptance limit of 0.30 in all cases, showing homogenous formulations. However, the formulation prepared at 60 °C showed significantly more negative zeta potential (−10.3 ± 1.8 mV) than the one at 70 °C (−8.1 ± 1.6 mV) (*, *p* < 0.05), while it did not differ from the 50 °C one (Figure 4A,B). Based on these data, 50–60 °C can be the design space for the liposomes with PC origin. According to this observation, 60 °C was chosen as the working temperature for the further studies presented in the article.

The DSC and TGA measurements of the liposome samples prepared from the same PC-CH composition at different temperatures (50, 60 and 70 °C) have resulted in the following curves: TG (black lines), dTG (red lines) and DSC (blue lines) diagrams. The dotted, full and dashed lines refer to the samples made at 50, 60 and 70 °C, respectively (Figure 4C). The results are congruent with the previous findings described in the literature. Based on the TG and dTG curves, the desorption of the physisorbed water content has been completed at around 100 °C for all the samples. The gel to liquid-crystalline phase transition temperatures (T_c_) of the samples made from the same compositions at different temperatures was ~30–32 °C, as the DSC measurements proved as well. Below the T_c_ value, the presence of the cholesterol makes the chains more mobile in the liposomes, preventing the hydrocarbon chains from crystallisation, which modifies the T_m_ and causes a separation before the phase transition—which is the reason why the curve is smooth—while above the Tc value it maintains the rigidity of the membrane [38]. Another change occurs at 200–225°, representing the molecular changes happening at this temperature range [39]. A total of 2–3% of the weight of the samples is lost during the heat treatment, which continued to 300 °C [40,41].

#### 3.3.2. Effects of Using Different Ratios of Wall-Forming Agents

**Figure 5:**
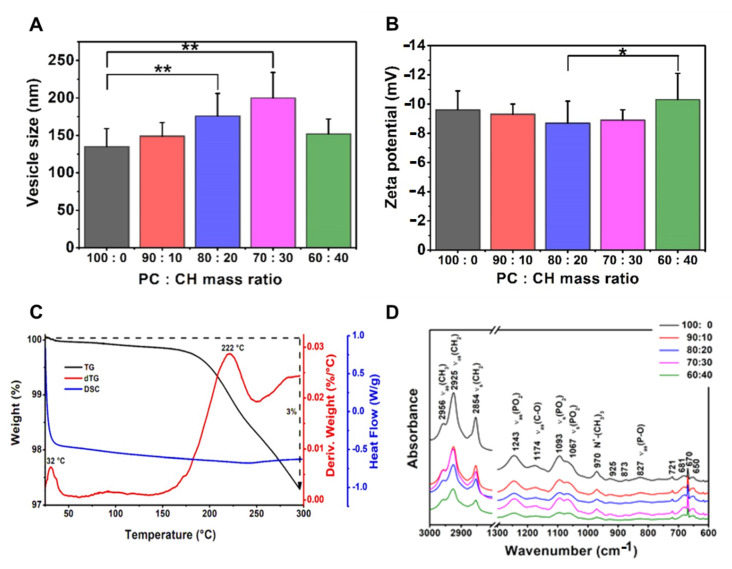
Characteristic features of the liposome samples prepared from different ratios of phosphatidylcholine (PC) and cholesterol (CH) at 60 °C, and hydrated with saline solution, presenting the results of the investigations: vesicle size and zeta potential analysis (**A**,**B**), differential scanning calorimetry and thermogravimetric analysis (**C**) and Fourier-transform infrared spectroscopy (**D**). *: *p* <0.05; **: *p* < 0.01.

The effect of using different phospholipid and cholesterol ratios was investigated in the PC-CH compositions prepared at 60 °C (Table 8, Figure 5). The size of the liposomes decreased with the reduction of the cholesterol ratio as the mass ratios changed from 70:30 to 100:0. The only PC-containing sample had significantly smaller vesicles (135 ± 24 nm) than the 20 *w/w*% (176 ± 30 nm) or 30 *w/w*% (200 ± 34 nm) CH-containing ones (**, *p* < 0.01). Applying lower proportions of phospholipids leads to larger vesicles until the 70:30 PC:CH mass ratio; however, the investigation of the PC:CH 60:40 vesicles indicated a decreased particle size (152 ± 20 nm). The lowest polydispersity index (0.18 ± 0.08) was measured in the same case as well. Our results strengthen the statement that the zeta potential values decrease by reducing the cholesterol concentration from the 30 *w/w*% content. This phenomenon may appear due to the presence of a higher number of phosphatidylcholine on the vesicle surface [42]. The most negative zeta potential value (−10.3 ± 1.8 mV) was measured in the case of the PC:CH 60:40 mass ratio liposomes, which significantly differs from the 80:20 samples (−8.7 ± 1.5 mV), (*, *p* < 0.05). Our results correlate with the work from López Pinto et al.; namely, an increment in the cholesterol concentration increases the size of the vesicles [43]. However, the cholesterol can maintain the rigidity of the liposomal membrane and improve its mechanical strength and packing density, thereby decreasing the permeability of water and small molecules through the membrane, as Magarkar et al. maintained in their work [44]. Based on these facts, cholesterol usage is recommended in the liposomal formulations to stabilise them; thus, in our case, the other investigations were carried out on cholesterol-containing compositions.

**Figure 6:**
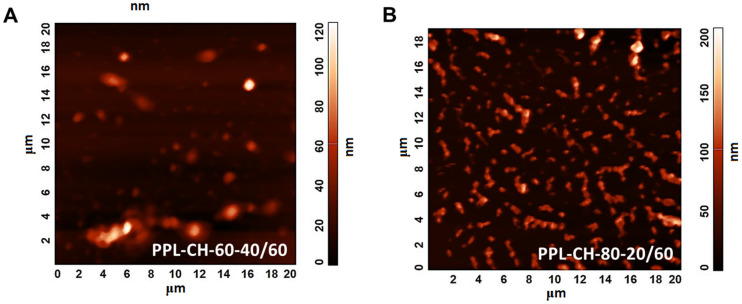
Atomic force microscopy images taken of the liposome samples prepared from PC-CH 60:40 (**A**) and 80:20 (**B**) mass ratio compositions at 60 °C, and hydrated with saline solution.

Figure 6 illustrates the AFM images of the PPL-CH-60-40/60 (Figure 6A) and PPL-CH-80-20/60 (Figure 6B) samples proving homogeneous size distribution and 120–150 nm of mean vesicle size consistently with the DLS results.

Figure 5C presents the DSC and TGA curves of the PPL-CH-60-40/60 liposome sample. As in all cases, the end of the physisorbed water content desorption was detected around 100 °C on the TG and dTG curves. The T_c_ temperature of the sample was 33 °C based on the DSC measurement result. The endotherm curve broadens with the rise of the cholesterol mole percentage; thus, the phase transition decreases [45,46]. Molecular alteration indicating changes are detected at the 200 225° range [39]. The thermal treatment causes a 3% mass loss in the samples until 300 °C [40,41].

The results of the FT-IR investigations made on the liposome samples prepared at 60 °C from different PC-CH compositions are shown in Figure 5D. The FT-IR spectra differ based on the type of the used lipids. In the PC-CH compositions, phosphatidylcholine (PC) is the wall-forming lipid, and the FT-IR figures showed two separate regions concerning the already known information about PC [47]. The so-called fingerprint region is at ~900–600 cm^−1^. The 3000–2800 cm^−1^ wavenumber domain shows the C-H stretching vibrations originated mainly from the hydrocarbon chains. The lower wavenumber region of the spectrum (below 1800 cm^−1^) belongs to the polar head groups of the phospholipids. The shape of the measured spectra was the same as those gained from the PC-CH 60:40 formulations produced at different temperatures (Figure 4D). At 827 cm^−1^ asymmetric ν_as_(P-O), at 970 cm^−1^ N+-(CH_3_)_3_, at 1067 cm^−1^ and 1093 cm^−1^ symmetric ν_s_(PO)_2_, at 1174 cm^−1^ asymmetric ν_as_(C-O) and at 1243 cm^−1^ wavelength asymmetric ν_as_(PO_2_), stretchings are detected, which is typical for the polar head groups [48]. The symmetric stretchings ν_s_(CH_2_) at 2854 cm and the asymmetric ones ν_as_(CH_2_) at 2925 and 2956 cm^−1^ are the characteristics of the apolar hydrocarbon chains [47]. The traces of the FT-IR curves were consistent despite the different sample production temperatures; all the investigated samples contained similar bonds.

#### 3.3.3. Effect of Using Different Concentrations of PEGylated Phospholipid

**Figure 7:**
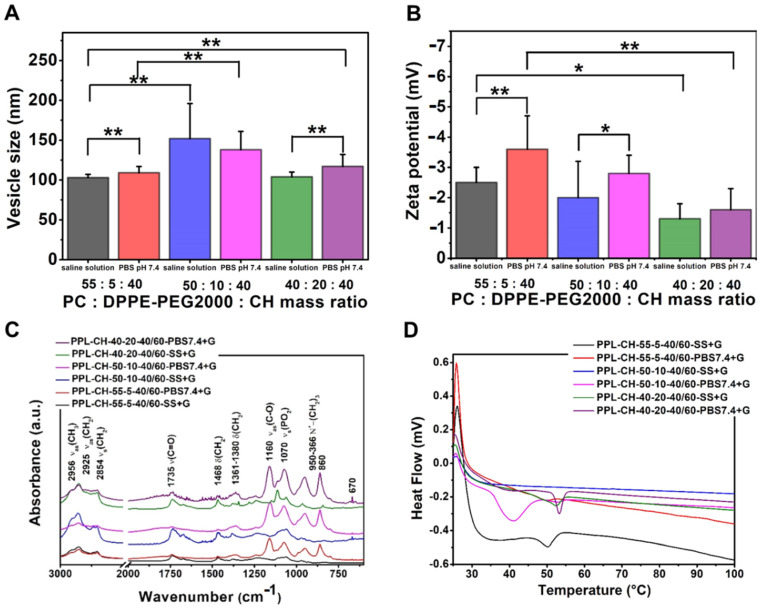
Characteristic features of the liposome samples prepared from different ratios of phosphatidylcholine (PC), PEGylated phosphatidylethanolamine (DPPE-PEG_2000_) and cholesterol (CH) at 60 °C, hydrated with saline solution or PBS pH 7.4, and lyophilised with glucose as cryoprotectant, presenting the results of the investigations: vesicle size and zeta potential analysis (**A**,**B**), differential scanning calorimetry (**C**) and Fourier-transform infrared spectroscopy (**D**). *: *p* < 0.05; **: *p* < 0.01.

The samples were made at 60 °C and hydrated with different media (saline solution and PBS pH 7.4) to study the effect of using different concentrations of PEGylated phosphatidylethanolamine. A non-linear relationship can be detected between the phospholipid ratios and the vesicle size (Table 9, Figure 7). The increase in the concentration of the PEGylated phospholipid from 55:5:40 mass ratio meant first larger vesicles (50:10:40 mass ratio), then a decrease in the size (40:20:40 mass ratio). The significantly largest particle size was measured in the case of the formulations made with PC:DPPE-PEG-_2000:_cholesterol 55:10:40 mass ratio for both hydration media (saline solution: 152 ± 44 nm; PBS pH 7.4: 138 ± 23 nm) (**, *p* < 0.01) (Figure 6A,B). Increasing the amount of the PEGylated phospholipids to this certain ratio enlarges the size of the vesicles. However, further addition causes a sharp decrease in the mean size value due to the formation of PEGylated phospholipid-based micelles, as Garbuzenko et al. described for disteroylphosphoethanolamine (DSPE)–PEG_2000_-containing vesicles [49]. Our results show the same phenomenon for DPPE-PEG_2000_ as well. The polydispersity index was measured as the lowest in the 55:5:40 case (0.25 ± 0.05; 0.20 ± 0.02). Thus, this formulation is the best regarding the particle size and uniformity, providing vesicles around 100 nm with uniform size. Even the zeta potential values were significantly more negative in case of the 55:5:40 ratios (−2.5 ± 0.5 mV; −3.6 ± 1.1 mV) than of the 40:20:40 ones (saline solution: *, *p* < 0.05; PBS pH 7.4: **; *p* < 0.01). Although the zeta potential values were negative, the highest was the used proportion of the DPPE-PEG_2000_; the least negative was the measured zeta potential value. Using PBS pH 7.4 resulted in moderately larger and more negative liposomes than those hydrated with saline solution.

Figure 7C presents the DSC measurements results of the PEGylated phospholipid-containing liposome. Remarkable phase transition can be observed in the case of the samples hydrated with PBS pH 7.4. This phase transition was detected at 52 °C for the PPL-CH-55-5-40/60-PBS7.4+G, at 40 °C for the PPL-CH-50-10-40/60-PBS7.4+G and at 50 °C in case of the PPL-CH-40-20-40/60-PBS7.4+G samples. The observed increase in the phase transition temperature originated from the decreasing lateral pressure as the hydrocarbon chains of PC and DPPE-PEG_2000_ became growingly mismatched as the membrane enriched with the PEGylated phospholipid [50].

The TG analysis (Figure 7C) resulted in curves resembling the previously described samples. The weight loss happened in two steps: first at 75–80 °C, then between 200–250 °C. The samples lost ~5% of their mass until 300 °C were achieved.

Figure 7D presents the results from the FT-IR measurements. The spectra were the same in the samples hydrated with PBS and saline solution independently from the composition ratios. Two different regions can be distinguished. The C-H stretching vibrations appeared in the 3000–2800 cm^−1^ wavenumber domain [47], while peaks typical to the polar head groups emerged below 1800 cm^−1^ in the lower wavenumber region: ester ν(C=O) at 1735 cm^−1^, δ(CH_2_) at 1468 cm^−1^, δ(CH_3_) between 1361–1380 cm^−1,^ ν(C-O) at 1160 cm^−1^ and ν(PO_2_) stretchings at 1070 cm^−1^ wavelength [48]. The lipid hydrocarbon chains can be detected in various spectral regions; however, the most significant ones appear between 3050 and 2800 cm^−1^. C-H stretching bands from different vibrational modes (ν_as_(CH_2_) at ~2917 cm^−1^ and ν_s_(CH_2_) at ~2850 cm^−1^) belong to this region. Some overlaps with other vibrations can be detected in this part of the spectra. Usually, these vibrational modes are uncoupled from the other modes; thus, they are not influenced by the lipid head groups but are sensitive to the structure of the chains [47].

**Figure 8:**
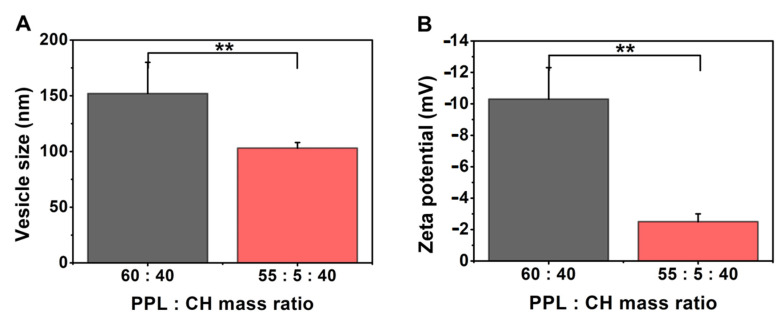
Characteristic features of the liposome samples prepared from phospholipid (PC only or PC and DPPE-PEG_2000_) and cholesterol (CH) at 60 °C, hydrated with saline solution or PBS pH 7.4 and lyophilised with glucose as cryoprotectant, presenting the results of the investigations: vesicle size and zeta potential analysis (**A**,**B**). **: *p* < 0.01.

Comparing the PEGylated phospholipid-containing liposomal formulations with the non-PEGylated ones (Table 10, Figure 8) led to the following observations. The addition of the PEGylated phosphatidylethanolamine to the PC-CH formulation (samples prepared at 60 °C and hydrated with saline solution) significantly decreased the size of the vesicles (**, *p* < 0.01), slightly increased the polydispersity of the samples and resulted in a significantly less negative zeta potential value (**, *p* < 0.01). Changing a part of the PC content to phosphatidylethanolamine (PE) decreases the size of the liposomes, as Akizuki and Kaneko showed in their work [51]. Our results show that even the usage of PEGylated PE could decrease the vesicle size. The reason behind this size-decreasing ability, according to Li et al., is that the bilayer structure can be stabilised by the application of non-bilayer lipids, such as the unsaturated PE [52]. Our finding that the addition of PEGylated phospholipids can decrease the size of the formulated vesicles agrees with the report by Tsermentseli et al. for DSPE-PEG_2000_ [53]. The significant decrease in the zeta potential value (**, *p* < 0.01) after the addition of the DPPE-PEG_2000_ is due to the positive charge of the PEGylated phospholipid [54].

#### 3.3.4. Effect of Using Different Types of Hydration Media

**Figure 9:**
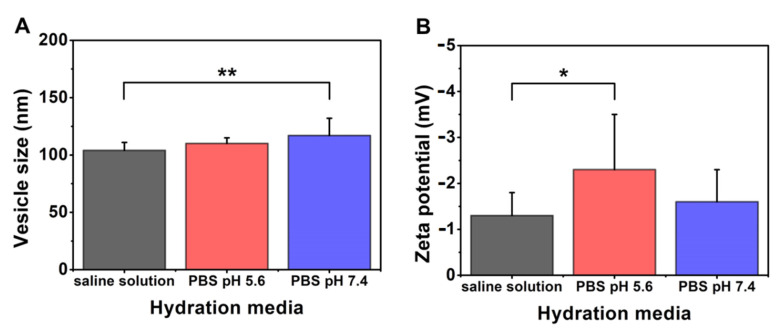
Characteristic features of the liposome samples prepared with different hydration media (saline solution, PBS pH 5.6 and 7.4) from PC-DPPE-PEG_2000_-CH 40:20:40 mass ratio composition at 60 °C, and lyophilised with glucose as cryoprotectant, presenting the results of the investigations: vesicle size and zeta potential analysis (**A**,**B**). *: *p* < 0.05; **: *p* < 0.01.

The effect of the quality of the hydration media on the PC-CH-PEG 40:20:40 mass ratio formulations made at 60 °C was studied (Table 11, Figure 9). The size of the particles increased in the order saline solution (104 ± 7 nm) < PBS pH 5.6 (110 ± 5 nm) < PBS pH 7.4 (117 ± 15 nm), with a significantly larger size in case of the PBS pH 7.4 than the saline solution (**, *p* < 0.01). The polydispersity of the formulations is mainly not influenced by the quality of the hydration media (0.29 ± 0.07; 0.33 ± 0.05; 0.26 ± 0.06), while a decrease could have been detected in the zeta potential values according to the following order: saline solution (−1.3 ± 0.5 mV) > PBS pH 7.4 (−1.6 ± 0.7 mV) > PBS pH 5.6 (−2.3 ± 1.2). Vesicles made with PBS pH 5.6 had significantly higher zeta potential than those hydrated with saline solution (*; *p* < 0.05). The ionic strength of the hydration media influences the value of the zeta potential; the higher the ionic strength is, the more compact the ion layer formed around the vesicles, and due to this phenomenon, the higher the zeta potential, as Tefas et al. described in their work [42]. In the presented case, the ionic strengths of the hydration media were: saline solution (0.15 M) < PBS pH 7.4 (0.16 M) < PBS pH 5.6 (0.40 M), which prove that the increasing ionic strength of the media increases the value of the zeta potential for the hydrated lipid vesicles. Therefore, not only its pH but also the ionic strength of the chosen solution should be determined for liposomal preparation.

Table 9 and Figure 7 show the compared results between saline solution and PBS pH 7.4 in the case of the two other liposome mass ratio compositions, PC-CH-PEG 55:5:40 and 50:10:40. The application of PBS pH 7.4 led to significantly larger vesicles not only for the 40:20:40 but for the 55:5:40 composition as well (**, *p* < 0.01). The zeta potential became significantly more negative after hydration with PBS pH 7.4 than with saline solution (55:5:40: **, *p* < 0.01; 50:10:40: *, *p* < 0.05).

**Figure 10:**
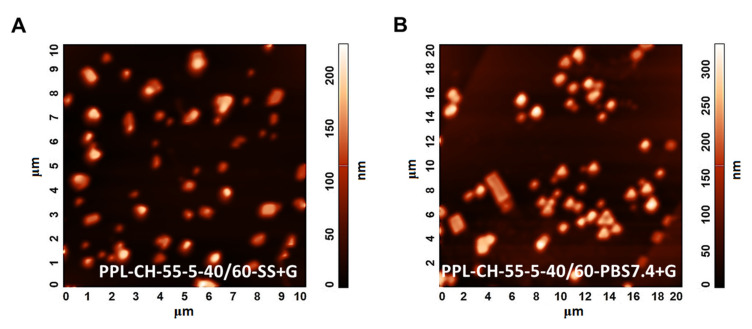
Atomic force microscopy images taken of the liposome samples prepared with different hydration media (saline solution (**A**), PBS pH 7.4 (**B**)) from PC-DPPE-PEG_2000_-CH 55:5:40 mass ratio composition at 60 °C, and lyophilised with glucose as cryoprotectant.

Figure 10 represents the AFM measurement results in the case of the PPL-CH-55-5-40/60-SS+G (Figure 10A) and the PPL-CH-55-5-40/60-PBS7.4+G (Figure 10B) samples, which differ only in the quality of the hydration media. The images show homogeneous size distribution and vesicles with 100–120 nm size, supporting the results of the DLS measurements.

#### 3.3.5. Effect of Using Different Types of Cryoprotectants

**Figure 11:**
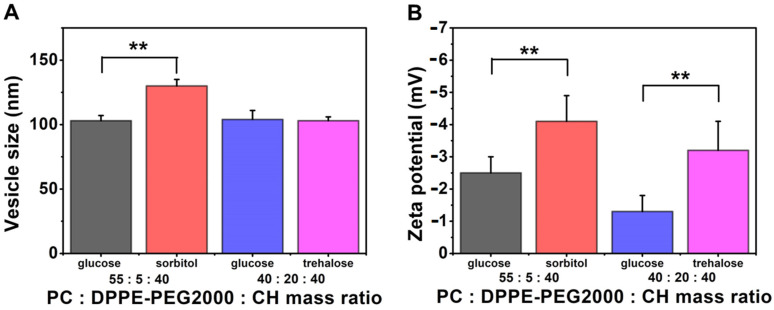
Characteristic features of the liposome samples lyophilised with different cryoprotectants prepared from PC-DPPE-PEG_2000_-CH 55:5:40 or 40:20:40 mass ratio composition at 60 °C and hydrated with saline solution, presenting the results of the investigations: vesicle size and zeta potential analysis (**A**,**B**). **: *p* < 0.01.

In total, 5% of the whole mass of the phospholipids was given to the hydration media from glucose, sorbitol or trehalose to investigate the difference between the effects of different cryoprotectants (Table 12, Figure 11). The PC-CH-PEG formulations made at 60 °C were used for this purpose. Both the 55:5:40 and the 40:20:40 PC:DPPE-PEG_2000_:cholesterol mass ratios were used for the study. When trehalose was used instead of glucose, the size of the vesicles was almost the same (103 ± 4 nm, 104 ± 7 nm, respectively). At the same time, sorbitol significantly increased the size of the liposomes (130 ± 5 nm and 103 ± 4 nm) (**, *p* < 0.01). The higher the ratio is of the DPPE-PEG_2000_ in the formulation, the less negative the zeta potential; however, it could have been determined that by using these cryoprotectants, the zeta potential values became more negative in the following order: glucose (−1.3 ± 0.5; −2.5 ± 0.5) > trehalose (−3.2 ± 0.9) > sorbitol (−4.1 ± 0.8). The sorbitol and the trehalose increased the zeta potential significantly compared to the glucose-containing formulations (**, *p* < 0.01 in both cases).

### 3.4. Residual Ethanol Measurements via Gas Chromatography-Mass Spectrometry (GC-MS)

Ethanol as a solvent has low toxic potential on the patients’ health according to the guideline of the ICH [55]. Class 3, to which ethanol belongs, includes no solvent known as a human health hazard at the commonly accepted levels in pharmaceuticals. A total of 50 mg per day or less of these residual solvents (corresponding to 5000 ppm or 0.5%) would be acceptable without justification. Even higher amounts can be accepted if they are realistic with regards to the manufacturing capability and good manufacturing practices. All the investigated samples (PPL-CH-60-40/60, PPL-CH-55-5-40/60-SS+G, PPL-CH-55-5-40/60-PBS7.4+G, PPL-CH-40-20-40/60-PBS5.6+G) contained ethanol under the limit of detection, showing safety towards the possible patients and the formulation itself.

## 4. Discussion

### 4.1. Development of the Knowledge Space, the Definition of the QTPP and the Identification of the CQAs, CMAs and CPPs

As a knowledge space development, the possible factors that can build up the quality target product profile (QTPP) for a liposome-based formulation, the general critical quality attributes (CQAs) of the liposomes were collected and the properties of the liposomes components and the thin-film hydration liposome preparation method were surveyed in a previous article [5]. This study narrowed the broad approach, and a stable, LUV-containing, monodisperse and homogeneous, API-free formulation was established as the QTPP of the “intermediate” liposomal products that can later provide suitable drug carrier systems (Table 5). The relevant CQAs, similar to the physical and chemical characteristics of the liposomes, were listed in Table 6. The CMAs and the CPPs regarding the indicated formulations were checked and compressed to assist the RA.

### 4.2. Risk Assessment and Design of Experiment

The collected knowledge was transformed in the LeanQbD^®^ software (QbD Works LLC, Fremont, CA, USA, www.qbdworks.com) into risk factors demonstrated with the severity scores during the RA. As the output, the former general liposomal RA has been refreshed, and the “intermediate” product-representing CQAs and CPPs were initiated with a numeric ranking reflecting their potential impact on the quality of the API-free formulations (Figure 2 and Figure 3). In the following, a DoE was set up to optimise the formulation process and inspect the correctness of the results. For this process, the factors with the highest risk severity scores were chosen from the CMAs and CPPs for experimental investigation. Five variables were identified, the working temperature (CPP), the phosphatidylcholine–cholesterol weight ratio (CMA), the PEGylated phospholipid content (CMA), the quality of the hydration media (CMA) and the quality of the cryoprotectants (CMA). A non-PEGylated (PC-CH, Table 1) and a PEGylated (PC-CH-PEG, Table 2) basic formulation were made up (Table 3) and then modified according to the levels of the investigated factors (Table 4). The effect of the working temperature and the mass ratio of the wall-forming agents was studied on the PC-CH composition. Based on the information obtained from these experiments, the effect of the PEGylated phospholipid content, the quality of the hydration media, and the cryoprotectant type were investigated under improved conditions (60 °C, PC-CH mass ratio 60:40) on the PC-CH-PEG formulations.

Our results, that the quantity and quality of the phospholipids and the cholesterol has the most significant impact on the final quality of the product, agrees with the findings in the work from Ahmed et al. [25], who investigated the question on glimepiride-containing phosphatidylserine-cholesterol liposomes. In the case of drug-loaded formulations, the API itself has a significant role in the liposomal properties, as it changes the structure of the liposome system [5,26]. Besides the drug-lipid ratio, Dhoble et al. studied the effect of the hydration and the sonication time on erlotinib-carrying 1,2-dipalmitoyl-n-glycerol-3-phosphocholine- and cholesterol-based liposomes [56]. They found that not only the composition but the sonication time has a significant impact on the entrapment efficiency.

### 4.3. Characterisation Results of the Liposomal Products

The working temperature is a rarely investigated parameter in the liposome preparation field. Our work showed that the size of the phosphatidylcholine–cholesterol (mass ratio 60:40) formulation is not affected by the temperature change; however, at 60 °C, significantly more negative zeta potential was reached than at 70 °C. The data suggest 50–60 °C as the optimal temperature for the preparation of these liposomes through thin-film hydration. The general practice suggests preparing liposomes above their transition temperature to receive efficient dispersion. The phase transition temperature of PC ranges between 50–55 °C [28,57]. Pandey et al. studied the formulation process of liposomes prepared via the ethanol injection technique in the temperature range of 40–70 °C. Their optimal working temperature range was 55–70 °C. In their case, the increase in the temperature resulted in insignificantly smaller particle size, which suggests that the formulation settings should be determined case-by-case for the different preparation methods. The effect of the changing phospholipid-cholesterol weight ratio was investigated at 60 °C, decreasing the CH content from 40% to zero. The size was decreasing with the reduction of the CH concentration. The size of the only PC-formed liposomes was significantly smaller than the 20% and 30% containing ones. López-Pinto et al. found the same correlation, that increasing the cholesterol concentration increases the size of the vesicles when investigated dipalmitoylphosphatidylcholine–cholesterol liposomes prepared according to the hydration technique [43]. After the concentration-based increase in the CH-content, significantly higher absolute zeta potential values were reached in the 40% CH-containing samples than in the 20% case, in parallel with some size reduction, which can strengthen the statement about the increasing effect of the cholesterol on the surface charge made by Sahu et al., who were screening this influence in the 20–50% of CH ratio [58]. As the CH maintains the mechanical strength of the membrane, it is suggested to complete the liposome compositions with cholesterol. In the following, 5–10–20% of the formulation weight was changed from PC to DPPE-PEG_2000_ to investigate the effect of the PEGylated phospholipid on the characteristics of the liposomes. The addition of 5% DPPE-PEG_2000_ to the PC-CH formulation caused a significant decrease in the size and the zeta potential of the vesicles due to the stabilised bilayer and the positive charge of the PEGylated phospholipid. Increasing the DPPE-PEG_2000_ concentration to 10% significantly enlarged the vesicle size; however, with further addition, the size dropped. A descending tendency was observed regarding the surface charge as well with the rise of the DPPE-PEG_2000_ concentration. Size and charge reduction were observed as well when DPPE-PEG_2000_ was added to linolenate-linolenic acid vesicles by Teo et al. [59]. The quality of the applied hydration media affects the characteristics of the liposomes as well. Vesicles made with PBS pH 7.4 (0.16 M) are significantly larger than those hydrated with saline solution. The zeta potential of the liposomes parallelly increases with the ionic strength of the hydration media due to the forming ion layer around the vesicles. In the investigated cases, hydration with PBS pH 5.6 (0.40 M) significantly enhanced the zeta potential of the vesicles in contrast to the saline solution (0.15 M). A similar tendency was noticed by Tefas et al. applying PBS pH 4.5 and 5.0 in the preparation process of curcumin-doxorubicin-hydrochloride co-encapsulating liposomes made from with 1,2-dipalmitoyl-sn-glycero-3-phosphocholine, N-(carbonyl-methoxypolyethyleneglycol-2000)-1,2-distearoyl-sn-glycero-3-phosphoethanolamine and cholesterol following the thin film hydration method [42]. In conclusion, the ionic strength of the hydration media should be considered when it is chosen for the formulation besides the required pH. Generally, 5% of the phospholipid mass used to be added to the formulations from carbohydrates as cryoprotectants. In this study, sorbitol caused a significant increase in the mean vesicle size compared to glucose and trehalose, while both sorbitol and trehalose increased the surface charge significantly. Hau et al. made quantitative observations about cryoprotectants on PC-CH liposomes [60]. The protective effect of various cryoprotectants is different. They suggest using 5% glucose, 10% sucrose, 15% mannitol, or 10% trehalose to achieve the best protective effect, as the smallest diameters for the vesicles were measured in these cases. They found that the mean vesicle diameter of liposomes prepared with glucose was the biggest, with trehalose as the smallest of these four carbohydrates. Sylvester et al. applied the QbD concept to understand the freeze-drying process more and found that combining the cryoprotectants (trehalose + mannitol) leads to better results [61]. Thus, consideration of the quality is suggested even for the cryoprotectants.

The thermal analysis has an important role in the characterisation of the liposomes due to the phase transitions of the phospholipids. The phase transition temperature is typical for the wall-forming lipids. Below the T_c_ value, the cholesterol mobilises the hydrocarbon chains hindering them from crystallisation that modifies the T_m_ and indicates a separation before the phase transition. In contrast, above the T_c_ value, cholesterol maintains the rigidity of the bilayer membrane [38]. Increasing the cholesterol content broadens the endotherm curve; thus, the observed phase transition lessens [45,46].

The physisorbed water content desorption ended around 100 °C in the case of all the investigated samples concluded from the TG and dTG curves. Another change could be detected in the shape of the curves at 200–225°, representing the molecular changes [39]. In total, 3–5% of the sample weight were lost during the heat treatment up to 300 °C [40,41].

The FT-IR spectra differ from type to type of wall-forming lipids. In the presented formulations, PC was the main component; thus, the FT-IR curves demonstrated two separate regions in the spectra that are typical for the phosphatidylcholine [47]: the fingerprint region was observed at ~900–600 cm^−1^, while the C-H stretching vibrations originated mainly from the hydrocarbon chains appeared in the 3000–2800 cm^−1^ wavenumber domain. The lower wavenumber region of the spectra (below 1800 cm^−1^) represented the polar head groups of the phospholipids. Differences among the FT-IR spectra of the samples hydrated with different hydration media were well-detectable. The typical υ_as_(PO_2_) and υ_s_(PO_2_) stretchings appeared in the case of the liposomes hydrated with the PBS solutions. The differing ionic strengths can cause the differences between the spectra of the samples hydrated with PBS of different pH. 

## 5. Conclusions

This QbD-guided and RA-based study aimed to determine the CMAs and the CPPs of an “intermediate”, API-free liposome formulation prepared via the thin-film hydration method and show the process of how to tighten a general initial RA for a specific case. The theoretical liposome design was followed by experimental modelling to prove the concept. The QTPP elements of these liposomal products were determined as spherical, large unilamellar vesicles (LUVs) in stable, homogeneous, monodisperse systems. The necessary CQAs that must be ensured to maintain the targeted QTPP were also collected parallel to the CMAs and the CPPs that need to be considered during the formulation design. How the screening of the factors should be done to find the most critical one is also presented. The quality of phospholipids, the quality and quantity of surface modifiers, the ratio between the phospholipids and the cholesterol, the type of the cholesterol, the phase transition temperature, the working temperature, the quality of the hydration media, the settings of the sonication, the quality and quantity of cryoprotectants, the properties of filtration, the sterility of the formulation, the quality of the solvent, the possible addition of other additives, the dissolution step for the lipids, the storage conditions, the settings of the lyophilisation and the vacuum evaporation were found as the most highly influencing CMAs/CPPs in descending order according to their severity scores. This RA result led to an effective practical experimental design to investigate the effect of the five riskiest parameters for an intermediate formulation (the working temperature, the phosphatidylcholine–cholesterol weight ratio, the PEGylated phospholipid content, the quality of the hydration media and the cryoprotectants). The prepared liposomes were investigated via the most typical analysation techniques. The characterisation findings (vesicle size, PdI, zeta potential, DSC, TGA and FT-IR) can support and complete the results of the updated RA. A working temperature of 50–60 °C might be ideal for the investigated PC-based formulations. Applying higher proportions of phospholipids leads to smaller vesicles. The increase in the ratio of PEGylated phospholipids enlarges the size of the liposomes, but further addition causes a decrease. From the investigated compositions, the PC-DPPE-PEG_2000_-CH 55:5:40 mass ratio fits the most to the formerly established criteria. The pH and ionic strength of the hydration media, as well as the type of the cryoprotectant, impact the liposomal formulation quality. The applied levels of the investigated factors and the number of the analytical techniques can be widened, and the compositions may be changed or completed even with APIs to meet the requirements of studies with more specific circumstances. In these cases, newly updated RAs should be established. RAs can be repeated and finalised in the later phases of the development cycle, when further or better information is available to assess the actual risk. This article aimed to provide a base for a practical decision-making system that facilitates modifying the features of the liposomes according to previously defined target values or criteria. The results of this study may help the researchers to perform a successful RA-based liposome design, set up the DoE and later a DS. 

## Figures and Tables

**Table 1:** Phosphatidylcholine and cholesterol-based (PC-CH) compositions.

Compositions	Phosphatidylcholine–Cholesterol Liposomes				
	Phospholipid:Cholesterol Mass Ratio				
	100:0	90:10	80:20	70:30	60:40
**PC (*w/w*%)**	100	90	80	70	60
**cholesterol (*w/w*%)**	-	10	20	30	40
	solvent of the stock solution				
**EtOH 96%**	+				
	hydration media				
**saline solution (mL)**	100				

**Table 2:** Phosphatidylcholine, PEGylated phosphatidylethanolamine and cholesterol-based (PC-CH-PEG) compositions.

Compositions	PEGylated Liposomes								
	PC:DPPE-PEG_2000_:Cholesterol Mass Ratio								
	55:5:40			50:10		40:20:40			
**PC (*w/w*%)**	55			50		40			
**DPPE-PEG_2000_ (*w/w*%)**	5			10		10			
**cholesterol (*w/w*%)**	40			40		40			
	solvent of the stock solution								
**EtOH 96%**	+								
	hydration media								
**saline solution (mL)**	100	-	100	100	-	100	-	100	-
**PBS pH 5.6 (mL)**	-	100	-	-	-	-	-	-	100
**PBS pH 7.4 (mL)**	-	-	-	-	100	-	100	-	-
	cryoprotectant								
**glucose (%)**	5	5	-	5	5	5	5	-	5
**sorbitol (%)**	-	-	5	-	-	-	-	-	-
**trehalose (%)**	-	-	-	-	-	-	-	5	-

**Table 3:** Nomenclature of the samples presented in the article.

**(A) Sample Name**	**Composition (m/m%)**						**Hydration Media**	**Cryoprotectant** **(5% of Total PPL. Mass)**
	**PC**			**CH**				
**PPL-CH-60-40/50-SS**	60			40			saline solution	-
**PPL-CH-60-40/60** **-SS**	60			40			saline solution	-
**PPL-CH-70-30/60-SS**	70			30			saline solution	-
**PPL-CH-80-20/60-SS**	80			20			saline solution	-
**PPL-CH-90-10/60-SS**	90			10			saline solution	-
**PPL-CH-100-0/60-SS**	100			0			saline solution	-
**PPL-CH-60-40/70-SS**	60			40			saline solution	-
**(B) Sample Name**	**Composition (m/m%)**						**Hydration Media**	**Cryoprotectant** **(5% of Total PPL. Mass)**
	**PC**		**DPPE-** **PEG_2000_**		**CH**			
**PPL-CH-55-5-40/60-SS+G**	55		5		40		saline solution	glucose
**PPL-CH-55-5-40/60-PBS7.4+G**	55		5		40		pH 7.4 PBS	glucose
**PPL-CH-50-10-40/60-SS+G**	50		10		40		saline solution	glucose
**PPL-CH-50-10-40/60-PBS7.4+G**	50		10		40		pH 7.4 PBS	glucose
**PPL-CH-40-20-40/60-SS+G**	40		20		40		saline solution	glucose
**PPL-CH-40-20-40/60-PBS7.4+G**	40		20		40		pH 7.4 PBS	glucose
**PPL-CH-40-20-40/60-SS+T**	40		20		40		saline solution	trehalose
**PPL-CH-40-20-40/60-PBS5.6+G**	40		20		40		pH 5.6 PBS	glucose
**PPL-CH-55-5-40/60-SS+S**	55		5		40		saline solution	sorbitol

**Table 4:** Critical factors and their levels investigated in the liposome formulation processes.

	Critical Factors	Investigated Levels or Parameters														
**C** **P** **P**	**working temperature**	50 °C					60 °C					70 °C				
**C** **M** **A**	**Phosphatidylcholine:cholesterol mass ratio**	100:0			90:10			80:20			70:30			60:40		
	**PEGylated phospholipid content** PC:DPPE-PEG2000:cholesterol mass ratio	5% 55:5:40					10% 50:10:40					20% 40:20:40				
	**quality of hydration media** pH ionic strength	saline solution pH 5.5 0.15 M					PBS pH 5.6 pH 5.6 0.40 M					PBS pH 7.4 pH 7.4 0.16 M				
	**quality of cryoprotectants**	glucose					sorbitol					trehalose				

**Table 5:** QTPP elements of the “intermediate” liposomes designed as optimal carrier systems.

QTPP Factors	Details	Comments/Justifications
**dosage form/** **appearance**	lyophilised powder	- stable solid powder - the dosage form can affect the potential administration routes and clinical application manners
**physical attributes**	morphology, large unilamellar structured, liposomes (LUV), optimal particle size, proper zeta potential	- the structure and the size of the vesicle is critically related to the potential dosage forms, dosage strength and administration routes, which can affect the incorporation of an API - critically related to excipients(e.g., surface modifiers and additives) - vesicles with a size of 100–200 nm can be suitable for several application methods
**stable structure**	in aqueous solution	- stability is a quality requirement; the duration of the stability is important - the stability of the formulation influences the safety, efficacy and quality profile of the product
	in freeze-dried powder form	
**homogeneoussystem**	homogenous formulation	- critically related to the quality of the later product - influenced by the polydispersity of the system

**Table 6:** CQAs of the “intermediate”, API-free liposomal formulation.

**CQAs**	**Details**	**Comments/Justification**
**type of liposomes**	conventional, cationic, immune, bioresponsive, magnetic	determine the quality of the lipids
**targeted delivery compatibility**	knowledge about the possible administration route	formulation needs to be suitable for the requirements of the later API
**size of the vesicles**	mean particle size: 100–200 nm	large vesicles (LUV)
**number of lamellas**	1 lamella	unilamellar vesicles (LUV)
**morphology**	shape and structure	spherical unilamellar vesicles
**polydispersity index (PdI)**	acceptable: below: 0.3	monodisperse system
**zeta potential**	the higher in absolute value, the more stable the formulation	indicates stability
**surface modifications**	attachment of polyethylene glycol (PEG) chains, monoclonal antibodies, antibody fragments peptides, nucleic acids, carbohydrates or small molecules	maintain targeted delivery
**specific surface area**	surface area-to-volume ratio	determines the properties of the later drug release
**phase transition temperature (T_m_)**	working temperature is recommended to be higher than T_m_	different value for each composition
**sterility**	meets the microbiological requirements	depends on the chosen administration route
**stability**	stable under given circumstances	in aqueous solution/in freeze-dried powder form

**Table 7:** Measurement results of the liposome samples prepared at different temperature values (50, 60 and 70 °C) from PC-CH 60:40 mass ratio composition and hydrated with saline solution.

Compositions	Phosphatidylcholine–Cholesterol Liposomes (Mass Ratio: 60:40)					
	50 °C		60 °C		70 °C	
	mean	SD	mean	SD	mean	SD
**vesicle size (nm)**	154	14	152	28	166	18
**PdI**	0.24	0.02	0.18	0.08	0.21	0.04
**zeta potential (mV)**	−9.6	2.0	−10.3	1.8	−8.1	1.6
**TG%**	2		3		2	
**sample name**	PPL-CH-60-40/50-SS		PPL-CH-60-40/60-SS		PPL-CH-60-40/70-SS	

**Table 8:** Measurement results of the liposome samples prepared from different phosphatidylcholine (PC) and cholesterol (CH) ratios at 60 °C and hydrated with saline solution.

Compositions	Phosphatidylcholine–Cholesterol Liposomes (60 °C)									
	Phosphatidylcholine:Cholesterol Mass Ratio									
	100:0		90:10		80:20		70:30		60:40	
	Mean	SD	Mean	SD	Mean	SD	Mean	SD	Mean	SD
**vesicle size (nm)**	135	24	149	18	176	30	200	34	152	20
**PdI**	0.24	0.02	0.25	0.03	0.26	0.03	0.30	0.08	0.18	0.08
**zeta potential (mV)**	−9.6	1.3	−9.3	0.7	−8.7	1.5	−8.9	0.7	−10.3	1.8
**TG%**	4		3		4		2		3	
**sample name**	PPL-CH-100-0/60-SS		PPL-CH-90-10/60-SS		PPL-CH-80-20/60-SS		PPL-CH-70-30/60-SS		PPL-CH-60-40/60-SS	

**Table 9:** Measurement results of the liposome samples prepared from different phosphatidylcholine (PC), PEGylated phosphatidylethanolamine (DPPE-PEG_2000_) and cholesterol (CH) ratios at 60 °C, hydrated with saline solution or PBS pH 7.4 and lyophilised with glucose as cryoprotectant.

Compositions	Cryoprotectant: Glucose; 60 °C											
	PC:DPPE-PEG_2000_:Cholesterol Mass Ratio											
	55:5:40				50:10:40				40:20:40			
	Hydration Media											
	Saline Solution		PBS pH 7.4		Saline Solution		PBS pH 7.4		Saline Solution		PBS pH 7.4	
	Mean	SD	Mean	SD	Mean	SD	Mean	SD	Mean	SD	Mean	SD
**vesicle size (nm)**	103	4	109	8	152	44	138	23	104	6	117	15
**PdI**	0.25	0.05	0.20	0.02	0.27	0.01	0.27	0.07	0.29	0.07	0.26	0.06
**zeta potential (mV)**	−2.5	0.5	−3.6	1.1	−2.0	1.2	−2.8	0.6	−1.3	0.5	−1.6	0.7
**TG%**	4		4		5		6		5		7	
**sample name**	PPL-CH-55-5-40/60-SS+G		PPL-CH-55-5-40/60-PBS7.4+G		PPL-CH-50-10-40/60-SS+G		PPL-CH-50-10-40/60-PBS7.4+G		PPL-CH-40-20-40/60-SS+G		PPL-CH-40-20-40/60-PBS7.4+G	

**Table 10:** Measurement results of the liposome samples prepared from phospholipid (PC only or PC and DPPE-PEG_2000_) and cholesterol (CH) at 60 °C, hydrated with saline solution or PBS pH 7.4 and lyophilised with glucose as cryoprotectant.

Compositions	Phospholipid:Cholesterol Mass Ratio 60:40 Liposomes; Saline Solution; 60°			
	PC: Cholesterol 60:40		PC:DPPE-PEG_2000_: Cholesterol 55:5:40	
	Mean	SD	Mean	SD
**vesicle size (nm)**	152	28	103	5
**PdI**	0.18	0.08	0.25	0.05
**zeta potential (mV)**	−10.3	2.0	−2.5	0.5
**TG%**	3		4	
**sample name**	PPL-CH-60-40/60-SS		PPL-CH-55-5-40/60-SS+G	

**Table 11:** Measurement results of the liposome samples prepared with different hydration media (saline solution, PBS pH 5.6 and 7.4) from PC-DPPE-PEG2000-CH 40:20:40 mass ratio composition at 60 °C, and lyophilised with glucose as cryoprotectant.

Compositions	PC:DPPE-PEG_2000_:Cholesterol Mass Ratio 40:20:40; Cryoprotectant: Glucose; 60 °C					
	Hydration Media					
	Saline Solution 0.154 M		PBS pH 5.6 0.40 M		PBS pH 7.4 0.16 M	
	Mean	SD	Mean	SD	Mean	SD
**vesicle size (nm)**	104	7	110	5	117	15
**PdI**	0.29	0.07	0.33	0.05	0.26	0.06
**zeta potential (mV)**	−1.3	0.5	−2.3	1.2	−1.6	0.7
**TG%**	5		15		7	
**sample name**	PPL-CH-40-20-40/60-SS+G		PPL-CH-40-20-40/60-PBS7.4+G		PPL-CH-40-20-40/60-PBS5.6+G	

**Table 12:** Measurement results of the liposome samples lyophilised with different cryoprotectants prepared from PC-DPPE-PEG_2000_-CH 55:5:40 or 40:20:40 mass ratio composition at 60 °C and hydrated with saline solution.

Compositions	Hydration Media: Saline Solution; 60 °C							
	PC:DPPE-PEG_2000_:Cholesterol Mass Ratio							
	55:5:40				40:20:40			
	Glucose		Sorbitol		Glucose		Trehalose	
	Mean	SD	Mean	SD	Mean	SD	Mean	SD
**vesicle size (nm)**	103	4	130	5	104	7	103	3
**PdI**	0.25	0.05	0.30	0.05	0.29	0.07	0.29	0.03
**zeta potential (mV)**	−2.5	0.5	−4.1	0.8	−1.3	0.5	−3.2	0.9
**TG%**	4		25		5		6	
**sample name**	PPL-CH-55-5-40/60-SS+G		PPL-CH-55-5-40/60-SS+S		PPL-CH-40-20-40/60-SS+G		PPL-CH-40-20-40/60-SS+T	

## Data Availability

MDPI Research Data Policies at https://www.mdpi.com/ethics.

## Abbreviations

AFM	atomic force microscopy
API	active pharmaceutical ingredient
CH	cholesterol
CMAs	Critical Material Attributes
CPPs	Critical Process Parameters
CQAs	Critical Quality Attributes
DLS	dynamic light scattering
DoE	Design of Experiments
DPPE-PEG2000	1,2-dipalmitoyl-sn-glycero-3-phosphoethanolamine-N-[methoxy(polyethylene glycol)-2000] (ammonium salt)
DS	Design Space
DSC	differential scanning calorimetry
DSPE–PEG_2000_	1,2-distearoyl-sn-glycero-3-phosphoethanolamine-N-[amino(polyethylene glycol)-2000] (ammonium salt)
dTG	derivative thermogravimetry
EMA	European Medicine Agency
FT-IR	Fourier-transform infrared spectroscopy
ICH	International Council for Harmonisation of Technical Requirements for Pharmaceuticals for Human Use
LUV	large unilamellar vesicles
PBS	phosphate-buffered saline
PC	L-α-phosphatidylcholine
PdI	Polydispersity Index
PE	phosphatidylethanolamine
PEG	polyethylene glycol
QbD	Quality by Design
QTPP	Quality Target Product Profile
R&D	Research and Development
RA	Risk Assessment
T_c_	gel to liquid-crystalline phase transition temperature
T_g_	glass transitions temperature
TGA	thermogravimetric analysis
T_m_	phase transition temperature
